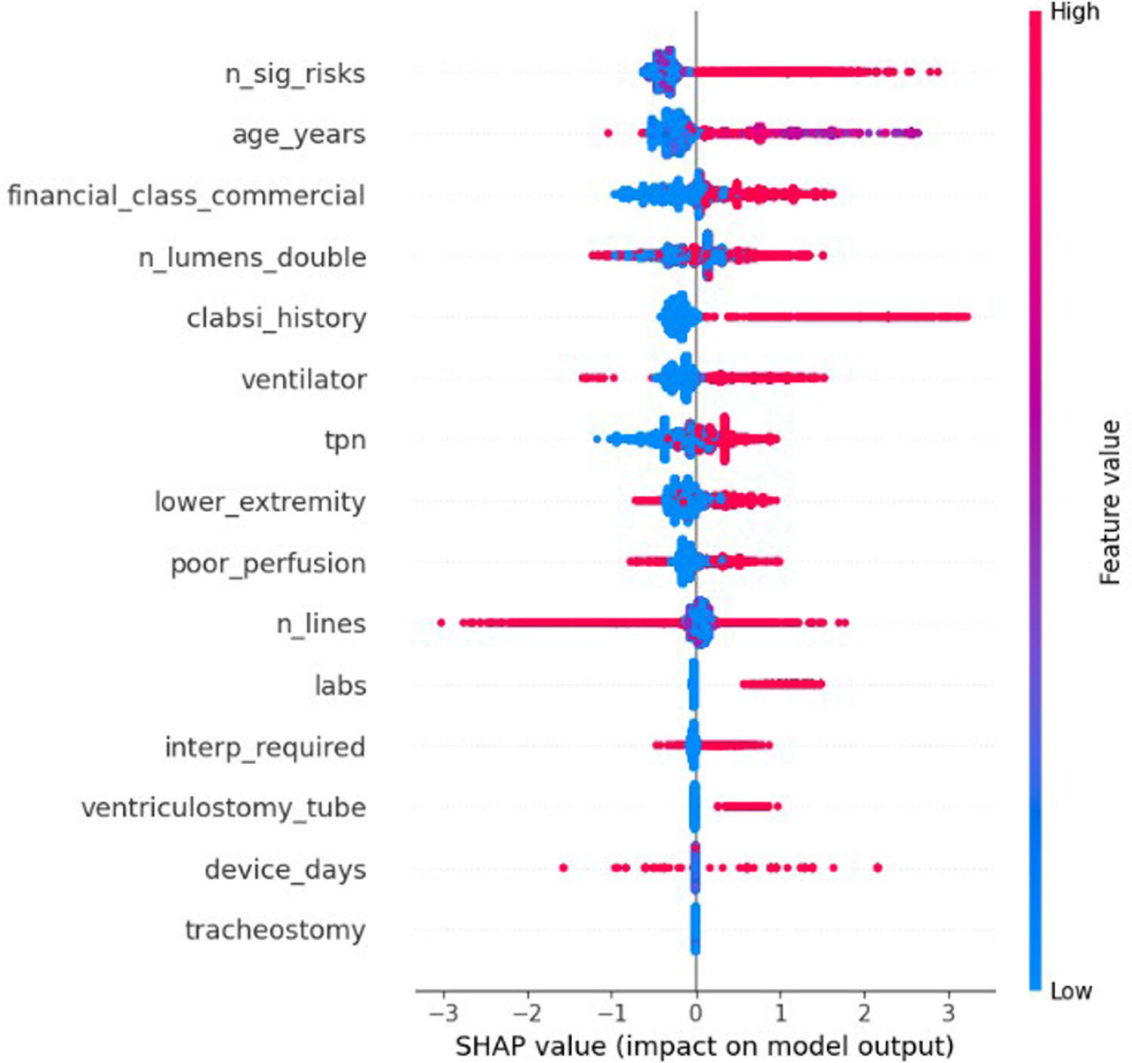# 315 Connecting the Patients: Network Analysis of a Multi-facility Candidozyma auris Outbreak, Georgia, September 2024 – August 2025

**DOI:** 10.1017/ash.2026.10736

**Published:** 2026-06-23

**Authors:** Erin Marshall, Michael Sayer, Christian Schneider, Lacey Bergerhofer, Azadeh Wickam

**Affiliations:** 1 Children’s Mercy; 2 Children’s Mercy Kansas City

## Abstract

**Background:** Central line-associated bloodstream infection (CLABSI) represents the most common and costly device-associated hospital-acquired infection in children. While prevention bundles are effective when reliably implemented, pediatric-specific risks often require strategies beyond standard bundles. Existing CLABSI risk models are insufficient for pediatric patients, remaining static during their hospital stay despite changing clinical conditions. Moreover, previously reported dashboards and predictive models generally do not incorporate data science principles to provide a daily prediction score, and they often exclude demographic factors beyond age and sex. The purpose of developing the CLABSI risk model was to leverage technology-driven strategies for identifying, intervening, and escalating care for high-risk pediatric patients in real time, thereby decreasing infection risk. **Methods:** Quality Improvement, Data Intelligence, and the interdisciplinary clinical team partnered to develop an evidence-based predictive model tailored to institutional risk factors. Using evidence and clinical expertise, an affinity diagram was created. Data from 61,068 inpatient central line days (January 2022- July 2024), sourced from electronic health records (EHR) and clinical surveillance software, formed the dataset. CLABSI was identified using NHSN surveillance definition. Patient risk factors from the infection date plus two days prior were evaluated. Exploratory data analysis was completed using RStudio integrated development environment to inform model development. Separate models were created for nontunneled lines and tunneled lines. Prediction models were developed using Python coding language and Databricks data intelligence platform. Under-sampling was used to account for uneven distribution between CLABSI and non-CLABSI cases. Logistic regression, random forest, and XGBoost models were created. Candidate models were compared using area under the curve (AUC), sensitivity, and specificity. **Result:** Using the XGBoost model for nontunneled line, the test AUC was 0.831, demonstrating strong discriminative power. This model achieved a sensitivity of 0.828, indicating its ability to correctly identify a high proportion of actual CLABSI cases, and a specificity of 0.751, reflecting its capacity to correctly identify non-CLABSI cases. Similarly, the tunneled line XGBoost model yielded an AUC of 0.845, showcasing excellent predictive performance. Its sensitivity was 0.833 and specificity was 0.668. In both models, the number of modifiable risks was the most significant factor (Tables 1-3). **Conclusion:** The CLABSI prediction model enables real-time identification and intervention to support proactive prevention, leveraging these performance characteristics to improve patient care. While predictive modeling has limitations due to EHR documentation, clinician expertise and frontline team engagement remain essential for deploying resources to highest risk patients.

**Figure:**
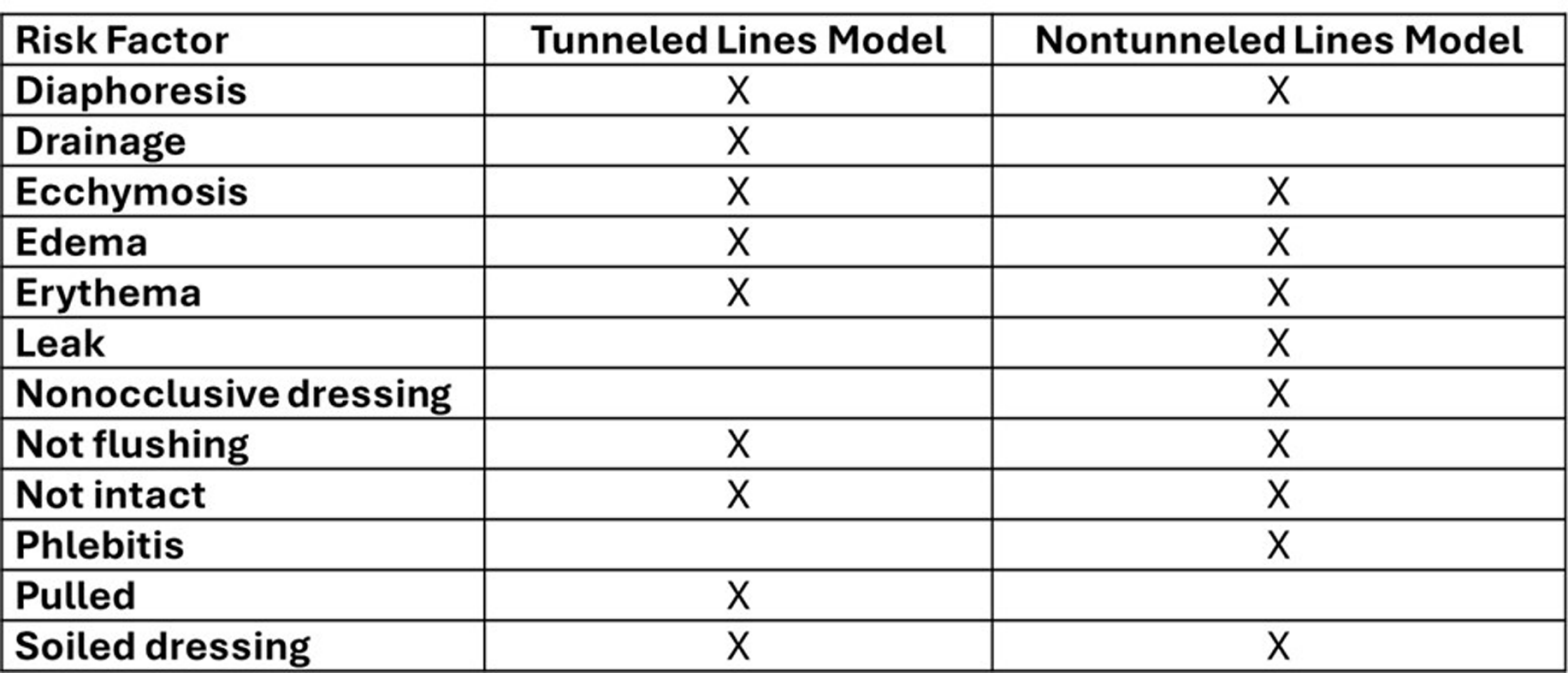

**Figure:**